# Leaves from banana (*Musa nana*) and maize (*Zea mays*) have no phyto-prophylactic effects on the susceptibility of grass carp (*Ctenopharyngodon idella*) to *Aeromonas hydrophila* infection

**DOI:** 10.1186/s12917-017-1255-5

**Published:** 2017-11-10

**Authors:** Richard Mayrhofer, Simon Menanteau-Ledouble, Johannes Pucher, Ulfert Focken, Mansour El-Matbouli

**Affiliations:** 1 Clinical Division of Fish Medicine, University of Veterinary Medicine, Vienna, Austria; 2 Life Science Centre, University of Hohenheim, Stuttgart, Germany; 3 Thünen-Institute of Fisheries Ecology, Ahrensburg, Germany

**Keywords:** Phytogenic supplement, Feed conversion ratio, Haematocrit, Diet composition, Stable isotope δ^13^C, Stress indicator

## Abstract

**Background:**

The ubiquitous and opportunistic bacterial pathogen *Aeromonas hydrophila* has been associated with ulcerative dermatitis in fish, especially under stressful conditions. It can cause severe losses in fresh water aquaculture and is particularly prevalent in tropical and subtropical regions. Fresh leaves from maize and bananas have been used as feed supplement by fish farmers in Vietnam and it has been reported that they may have phyto-prophylactic benefits. In the present study, a feeding trial was conducted to investigate the benefits of providing maize and banana leaves as feed supplement: to determine if they were taken up and digested by grass carp (*Ctenopharyngodon idella*), if this uptake resulted in improved growth performance, and if leaf supplementation protected fish when challenged with *A. hydrophila* by intramuscular injection.

**Results:**

All fish were fed an identical ratio of commercial pelleted feed relative to biomass. However, in 12/18 tanks, this diet was supplemented with either fresh banana leaves or fresh maize leaves; offered ad libitum. Addition of leaves increased the overall feed conversion ratio (FCR) significantly. However, if only the pellet were taken into account, then no difference was found between treatments. Changes to the isotopic composition of the fish showed leaf nutrient uptake occurred. No prophylactic effects of feeding banana or maize leaves were detected against infection with *A. hydrophila*, and the diet did not induce changes in the fish haematocrit. However, addition of the maize leaves was associated with significantly reduced severity of the skin lesions, which could improve the market value of the fish.

**Conclusions:**

Addition of the leaf supplement did not result in significantly improved growth performance. Similarly, the effect of the supplement on the fish survival to infection was not significant.

**Electronic supplementary material:**

The online version of this article (10.1186/s12917-017-1255-5) contains supplementary material, which is available to authorized users.

## Background

The macro-herbivorous grass carp (*Ctenopharyngodon idella*) is one of the most cultivated fish species worldwide. It is often stocked as the main species in semi-intensive polyculture systems with other fishes [1]. This practice enables small-scale farmers to produce fish for food security and sale at local markets, particularly when using leaves from other farming activities as low-cost feed [1]. However, Steinbronn et al. [1] report the occurrence of mass mortalities associated with grass carp-specific diseases in the ponds. Such outbreaks have the potential to strongly reduce the profitability of this aquaculture practice.

*Aeromonas hydrophila* is a ubiquitous, opportunistic bacterium that has been associated with high morbidity and mortality in fish, and is particularly problematic at higher temperatures [2], which are typical of regions where grass carp are farmed [3]. In aquaculture, the most common treatments against bacterial diseases remain chemotherapeutic and antibiotic agents which, when applied indiscriminately, have been linked to the development of drug resistant bacteria [4, 5]. Moreover, these treatments are often not available or affordable for small-scale farmers in rural areas like in Northern Vietnam. Alternatives to the application of antibiotics have therefore been investigated, including phytogenic feed supplements, several of which have been shown to improve resistance to bacterial infection [6–9]. In Asian grass carp-based polyculture systems, leaf material is a major feed input while in small-scale aquaculture, plant-based by-products from other farming activities are used as feeds. Among these, banana leaves are available year-round and are commonly supplemented to fish ponds [1, 10, 11]. In *Clarias gariepinus* larvae, banana leaves have been reported to reduce mortalities and improve growth and weight gain parameters [12].

Maize (*Zea mays*) leaves are similarly sporadically added to fish ponds, although less commonly than banana leaves as they are only available during part of the year [10]. While dried maize leaves were shown to be poorly digested by grass carp and have a negative effect on the digestibility of the rest of the diet, fresh maize leaves appeared to be well digested and were a promising supplement to the feed of grass carp [13].

Accordingly, we undertook this study to evaluate the consequence of adding fresh banana and maize leaves to the diet of grass carp. We analysed the isotopic signature of fish to determine if phytonutrients were assimilated by the carp. We assessed effects of the leaves on the growth performance of the fish and if they afforded any prophylactic effect against infection with the bacterium *A. hydrophila.*

## Results

### Growth performance and composition of feeds and fish carcasses

The feed materials used in this experiment differed in their chemical composition (Table 1): Maize leaves had a higher crude protein (CP) content than banana leaves: 27.9 and 20.0% of the total dry matter (DM) content, respectively, while the crude lipid (CL) contents were comparable between the two leaves (5.4% in the banana leaves and 4.9% in the maize leaves; Table 1).

**Table 1:** Chemical composition and isotopic composition of the lipid and lipid-free fraction of the feeds and grass carp analysed during the experiment

	Pellets	Banana Leaves	Maize	Fish fed the un-supplemented pellets		Fish fed the Banana leaves diet		Fish fed the Maize leaves diet	
				Non-infected	Infected	Non-infected	Infected	Non-infected	Infected
Dry Matter [% FM]	92.6	22.2	25.7	32.3 ± 1.2	31.1 ± 1.4	30.1 ± 1.0	30.0 ± 1.2	30.7 ± 0.7	31 ± 0.8
Crude Ash [% FM]	7.6	9.3	12.5	9.5 ± 0.5	9.5 ± 0.7	10.4 ± 0.7	10.5 ± 1.1	10.1 ± 0.5	9.7 ± 0.2
Crude Protein [% FM]	43.5	20.0	27.9	43.5 ± 0.8	45.7 ± 1.4	45.3 ± 5.4	48.0 ± 3.3	50.1 ± 4.5	49.9 ± 8.6
Crude Lipid [% FM]	8.3	5.4	4.9	39.7 ± 1.3	39.1 ± 2.4	35.0 ± 2.4	34.5 ± 3.9	36.0 ± 3.2	36.7 ± 2.0
Gross Energy [Mj kg^−1^ DM]	19.7	19.7	18.4	26.7 ± 0.3	26.6 ± 0.6	26.1 ± 0.2	25.7 ± 0.7	25.5 ± 0.6	26.0 ± 0.4
δ ^13^C/^12^C in lipid fraction	−26	−30.7	−20.6	−27.3 ± 0.0	−27.4 ± 0.1	−27.5 ± 0.1	−27.6 ± 0.0	−26.7 ± 0.1	−26.8 ± 0.1
δ ^13^C/^12^C in lipid-free fraction	−24.7	−26.7	−12.4	−22.8 ± 0.0	−22.8 ± 0.0	−23.0 ± 0.0	−23.0 ± 0.1	−21.8 ± 0.2	−21.6 ± 0.2
δ ^15^N/^14^N in lipid fraction	62.7	49.9	30.6	84.9 ± 14.1	127.8 ± 1.2	90.4 ± 20.0	87.9 ± 12.1	79.0 ± 16.3	113.0 ± 35.5
δ ^15^N/^14^N in lipid-free fraction	4.8	7.7	4.0	7.9 ± 0.1	7.8 ± 0.1	7.9 ± 0.2	7.9 ± 0.2	8.2 ± 0.1	8.0 ± 0.3

Three different feeding regimes were applied as well as bacterial infection with *Aeromonas hydrophila*. In total, 18 groups were included and each combination of feeding group and infectious status (infected and mock infected) was represented by three tanks. The values in this table represent the mean and standard deviation for each parameter

Supplementation of the feed with leaf material did not significantly affect the DM, Crude Ash (CA), and CP of the fish (Table 1). Significant effects on the CL content and gross energy (GE) were detected with higher CL content and GE in the fish that were solely fed pellets (39.4 and 26.7% respectively) compared to the fish that had received either the feed supplemented with banana leaves (average CM and GE values of 34.8 and 25.9%, respectively) or the fish that had received the maize leaves supplement (36.4 and 25.7%, respectively; Table 1).

The isotopic compositions of feeds differed in their δ^13^C and δ^15^N values (Table 1). The leaves of banana and maize showed strongly different δ^13^C values in both the lipid and the lipid-free fraction with higher δ^13^C signatures in the C_4_-plant maize. In the lipid-free fraction, banana leaves and pellet feed had a comparable signature of carbon isotopes (22.8 and 23, respectively). The lipid fraction of banana leaves showed the lowest δ ^13^C value compared with the lipid-free fraction, and with the other feed materials (Table 1). Lipid fractions showed significantly higher δ^15^N than the lipid-free fractions, with highest δ^15^N in de-fatted pellet feed followed by banana and maize leaves (Table 1). The lipid-free fraction of banana leaves had a higher δ^15^N than the lipid-free fractions of maize leaves and pellet feed (Table 1).

The different feeding regimes had a significant effect on carbon isotopic signature of grass carp (Table 1) with significantly higher δ^13^C values in the lipid and lipid-free fractions of fish fed fresh maize leaves (Table 1).

Bacterial infection affected neither the growth of the fish nor their chemical composition, but was associated with a significantly reduced ^13^C value in the fish lipids regardless the feeds. Infection also correlated with higher δ^15^N in the lipid fractions of grass carp (113 compared with 79; Table 1).

The average weight gain did not differ significantly between treatments (*p* = 0.27): 74.3 g in maize-supplemented fish,73.2 g in banana-supplemented fish, and 62.3 g in unsupplemented fish (Table 2). In all tanks, all pellet feed was consumed every day. The feed conversion ratios (FCR) were calculated both with and without taking the amount of leaf supplements into account (Table 2). When including leaves, the FCR values were higher in the fish that have received both the maize and banana supplement (5.20 and 4.27, respectively) than in un-supplemented fish (2.87, Table 2). These differences were significant between each feed group, including between the fish that had received the feed supplemented with maize and banana (*p* = 0.007).

**Table 2:** Farming performance of the fish during the experiment

	Fish fed the pellets without supplement		Fish fed the Banana leaves diet		Fish fed the Maize leaves diet	
	Non-infected	Infected	Non-infected	Infected	Non-infected	Infected
Weight gain	74.0 ± 11.8	50.5 ± 14.9	77.7 ± 13.1	70.8 ± 19.1	75.8 ± 14.0	70.6 ± 9.8
FCR (supplement excluded)	3.2 ± 0.7	2.5 ± 0.4	2.35 ± 0.15	2.20 ± 0.27	2.32 ± 0.19	2.25 ± 0.29
FCR (supplement included)	–	–	5.44 ± 0.40	4.95 ± 0.42	4.22 ± 0.24	4.32 ± 0.73
Average daily supplement consumption relative to total biomass (%)	–	–	4.44 ± 0.04	0.49 ± 0.03	0.71 ± 0.02	0.69 ± 0.01
Protein efficacy ratio (including supplement)	1.0 ± 0.1	0.8 ± 0.2	1.0 ± 0.1	1.0 ± 0.1	0.9 ± 0.1	0.9 ± 0.1
Specific growth rate	0.22 ± 0.01	0.17 ± 0.01	0.23 ± 0.01	0.25 ± 0.01	0.23 ± 0.00	0.25 ± 0.01

In total, 18 groups were included and each combination of feeding group and infectious status (infected and mock infected) was represented by three tanks. The values in this table represent the mean and standard deviation for each parameter

If the amount of leaves was not taken into account, the FCR was 2.27 in the fish that had received maize and 2.29 in the fish that had received banana (Table 2). These were not significantly different from 2.87, the value of the FCR from the fish that had received the un-supplemented feed (*p* = 0.051 and *p* = 0.057, respectively). Similarly, no statistically significant differences were observed between the feed uptake of infected and non-infected fish (*p* = 0.95 for the total feed uptake and *p* = 0.31 for the uptake of pellet feed).

The average daily intake of supplement represented 0.46 and 0.70% of their biomass for the fish that had received banana leaves and maize leaves, respectively (Table 2, Additional files 1 and 2). There was a significant difference in the quantity of supplement eaten: fish fed maize leaves ate more than fish fed banana leaves (*p* = 0.02). There was no statistically significant difference in the leaf consumption of infected versus non-infected fish (*p* = 0.82). Protein efficiency ratios (PER, Table 2) were not statistically different between the three treatments. Similarly, the infection did not have any significant effect on either FCR or PER. Conversely, the specific growth rate (SGR) was significantly lower in fish that had only received pellets compared with fish that had received either banana (*p* = 0.035) or maize leaves (*p* = 0.03).

### Disease susceptibility

The haematocrit levels were similar and no statistically significant differences were found between feeding groups (*p* = 0.241): 36.40 in pellet-only fish; 35.93 in maize-supplement fish; 35.31 in banana-supplement fish (Table 3, Additional file 3). Moreover, the infection status did not significantly affect the haematocrit levels of the fish (*p* = 0.285).

**Table 3:** Summary of the results from the challenge experiment. The values in this table represent the mean and confidence intervals for each parameter

Feeding group	Cumulative mortality [% of fish]	Average haematocrit [%]	Bacterial load in dead fish [CFU/ml]	Average grade of the skin lesions
Pellets	10.0 ± 3.89%	36.40 ± 4.10	9.23^+05^ ± 5.88^+06^	1.73 ± 0.33
Pellets + banana leaves	26.7 ± 5.95%	35.31 ± 2.99	9.90^+07^ ± 9.31^+07^	1.20 ± 0.27
Pellets + maize leaves	10.0 ± 0.0%	35.93 ± 3.06	3.06^+07^ ± 2.22^+07^	0.93 ± 0.21

Bacterial loads where calculated from the head kidney of fish that died following the challenge with *A. hydrophila*. The severity of the skin lesions was estimated individually for each based on the criterion by Lio-Po et al. [20]. Data are shown as the mean and standard deviation, as calculated for each infected fish that had received the same feeding regimen. Each of these feeding regimens was represented by three tanks

Following challenge with *A. hydrophila*, mortality occurred within 48 h following infection. The levels of mortality were not significantly different (*p* = 0.181) between the fish that had been fed either the pellet or the pellet supplemented with maize leaves (Table 3) and no mortalities were observed in the fish that had not been challenged.

Only infected fish developed skin lesions. On average, these lesions appeared more severe in the fish that had only received the pellet feed, with an average score of 1.73 based on the system described by Lio-Po et al. [14]. The average score was 1.20 in the fish that had received the banana leaves, and 0.93 in the fish that had received the maize leaves (Table 3), which were significantly lower than in control fish (*p* = 0.044). Interestingly, ulcerative lesions were rarely severe on fish that had died of the infection and generally scored lower than the lesions from fish that recovered.

The head kidneys of all fish were sampled for bacteria, but *A. hydrophila* could only be isolated from fish that had died of the infection. No bacteria were isolated from fish that were either not challenged or that survived to termination at 10 d after infection. When present, bacterial loads were 6.5 × 10^6^ CFU (colony forming units) per ml of head kidney. The differences between the bacterial loads of the three groups were not statistically significant (*p* = 0.784, Table 3).

## Discussions

### Feed consumption and absorption and growth parameters

Maize leaves were eaten significantly more than banana leaves (*p* = 0.02), possibly due to a difference in palatability for the fish. Despite being offered ad libitum, supplemental leaf materials were consumed at only low levels (below 1% of fish body mass per day). It has been reported that uptake of leaf material by grass carp is temperature dependent, and between water temperatures of 22–33 °C, grass carp can ingest up to 100% of their body mass in leaf material per day [15]. The temperature during this study was within this range and a higher feed intake could have been expected, which suggested that the application of pellet feed at 4 g kg^-0.8^ probably reduced the uptake of leaf material.

The banana leaves used in the present study contained a slightly higher CP compared to the composition reported from rural Northern Vietnam. On the other hand, the CL content was comparable to that previously reported [1, 10, 11]. The maize leaves that were used in this trial had a higher CP and CL content than that previously reported from rural Northern Vietnam [1]. However, the authors of the previous report sampled the entire material that was added to the pond without determining if only parts of the leaves were consumed. The composition of maize leaves in the present study was similar to the palatable fraction from maize leaf from rural Northern Vietnam, as reported by Pucher et al. [11] who analysed separately the plant parts that were palatable and non-palatable to grass carp. Fresh maize leaves are more digestible to grass carp than dried leaves. Dried leaves are less digestible and can negatively affect the growth of fish [13].

Steinbronn et al., reported FCRs of 7.7 for plant dry matter and 7.0 for plant fresh matter, after exclusively feeding leaf material to grass carp under field conditions [1]. In our study, the lower nutritional quality of supplemental leaves resulted in significantly higher FCRs when the weight of the leaves was added to that of the pelleted feed for the calculation.

In rural areas in Asia, the cost and availability of artificial feed is very different from that of the leaves, which are a by-product of local agricultural activities and can be acquired at low or no cost. We therefore calculated the FCRs without including the weight of the leaves, as this is more representative of the impact of using leaves on the farmer’s income. This later calculation did not show any significant difference in FCR between control and leaf-fed fish in either the maize trial (*p* = 0.051) or banana trial (*p* = 0.057). The PER was also similar between treatments (Table 2) while the SGR was lower in the control fish compared to those that received the leaf supplements. Intriguingly, this appears due to the lower SGR of infected fish that had not received the leaf supplement. If only the mock infected fish were considered, the difference was no longer significant (*p* = 0.77). While this suggests there was a protective effect of the leaf supplement, this hypothesis is not supported by the fact that few other parameters were affected by leaf supplementation.

Nutrient assimilation from feed to fish can be tracked using feeds with different isotopic signatures Schroeder [16, 17] used naturally occurring differences in carbon isotopes (δ^13^C) between natural food resources from the pond environment and external feeds from terrestrial or marine environments, to estimate the sources of nutrients for fish growth. This approach was further developed by separating lipid and non-lipid components in the fish diets [18, 19]. We used this approach to show that the leaf material contributed to the growth of the fish. Changes in δ^13^C and δ^15^N values has been correlated to bacterial infections in humans and used as a diagnostic tool, for example in *Helicobacter pylori* [20]. We observed that that infection induced changes in the isotopic signatures of the lipid fraction, even in the absence of outward signs of infection. This is the first observation of this kind in cyprinid fish, and we consider that this approach might be developed as an indicator for stress in the life history of fish.

### Haematocrit and disease resistance

We observed that leaf supplementation had no significant effect on the fish haematocrit (*p* = 0.241) or on resistance against *A. hydrophila* (*p* = 0.236). The average severity of the skin lesions per fish, determined according to Lio-Po et al. [14], was highest in fish that received only pellets and lowest in fish that had received the maize leaf supplement (*p* = 0.044). These results suggest that maize leaves might have had a beneficial effect on the fish during the infection, as has been shown for other phytogenic compounds in fish [6]. Our observations are the first to suggest that maize leaves may reduce the severity of skin lesions caused by *A. hydrophila* infections. Maize-leaves extracts have been shown to have antioxidant properties [21], as do maize flowers and cobs [22, 23]. Thus these antioxidant properties might have a protective effect against bacterial pathogens, and reduce scarification while having little effect on clearing of the bacteria. Indeed, studies by Wahli et al. [24] have shown that vitamin C has a positive influence on wound healing. While the concentration of vitamin C in maize leaves is not known, the antioxidants known to be present [21] might play a similar role specifically in wound healing. This would explain why the reduction in the severity of the lesions did not correlate with any improved survival. Because leaf supplement was distributed in addition to the pellet feed, it is possible that the limited protection observed was due to the supplemented diet containing more energy and nutrient. However this hypothesis was not supported by any difference in the growth parameters of the fish supplemented versus non-supplemented fish group, which would be expected if the nutrient values of the two diets were different. Changes in isotopic composition of the fish indicated that leaf nutrients were assimilated; however their lower energy content relative to pellet feed may have negated any measurable difference in growth performance between leaf supplemented and non-supplemented groups.

Severity of lesions was lower in fish that died of the infection, which suggests that septicaemia and death occurred too rapidly (< 48 h) for the fish to develop clinical signs. No bacteria were isolated from the kidneys of fish that had survived 10 days after infection. However, several of these fish did show skin lesions, which indicated that infection had taken place. We infer that either the bacteria were never able to develop a systemic infection in these fish or that the fish immune system had cleared the pathogen within 10 days.

It is noteworthy that no significant difference were found in the feed uptake of infected versus non-infected fish. This suggests that, for most fish the infection was not severe enough to interfere with appetite. Similarly, infection did not significantly affect the haematocrit levels of the fish (*p* = 0.285), which is consistent with the findings of Li et al. [25].

## Conclusions

The supplemental feeding of fresh maize and banana leaves did not significantly increase the growth performance of grass carp, which attests to the poor nutritional value of these types of plant materials. We demonstrated that the isotopic signatures of the feeds, in particular the isotopic signature of carbon from the leaves, were a suitable tool to confirm the assimilation of nutrients into fish biomass. Bacterial infection of grass carp was found to alter the isotopic composition of the lipid fraction of fish, even in the absence of strong clinical signs of bacterial infection. However, this effect needs additional investigation to assess its potential diagnostic use as a diagnostic technique.

The addition of fresh banana and maize leaves to the diet of grass carp did not result in a significant phyto-prophylactic effect against *A. hydrophila*: it did not significantly affect mortality or the haematocrit of the fish. However, fish that had received maize-leaf supplementation developed significantly less severe skin lesions than fish on un-supplemented feed.

## Methods

### Fish maintenance, feed utilization and growth performance

Clinically healthy grass carp (*N* = 180; Austrian Teichwirtschaft Gut Waldschach I) with no signs or history of *A. hydrophila* infection, and length ~17 ± 2 cm were randomly assigned to 18,100 L tanks (10 fish per tank) with flow-through water at 23 ± 3 °C, with at least 20% water exchange rate per day and a constant supply of oxygen. The fish remained in these tanks and no fish were moved during the duration of the experiment. The average biomass in each tank rose from 6.7 kg fish/m^3^ at the start of the experiment to an average of 13.7 kg/m^3^. All fish groups were fed full pellet feed (Garant-Tiernahrung GmbH) at a daily level corresponding to ~0.9% of the fish biomass. Six groups received fresh banana leaves and another six groups received fresh maize leaves distributed ad libitum as supplemental feed while the remaining six groups did not receive and supplemental feed. To prevent overfeeding, the fish were not fed for one day every week. The faeces and leftover feed were removed daily. The concentration of nitrite and ammonia were checked daily to confirm that they were below 5 and 20 PPM, respectively.

Fish remained under these conditions for 40 weeks, to mimic the field conditions in Vietnam. They were weighed every 2 wk. together rather than individually to minimize stress. The amount of pellet fed was adjusted to the weight of the fish. The weights of both eaten pellet feed and leaf material were recorded and feeding regimen calculated. At the end of the experiment, 10 d after the bacterial challenge, the total of the pellet feed and supplemental leaf material eaten, and fish weight gain were calculated and compared to the amount of feed distributed to calculate the FCR, with and without taking into account the amount of fresh leaves distributed. PER was calculated by the formula: fish fresh weight gain divided by amount of crude protein fed in pellet feed and supplemental feed. SGR was calculated based on the formula described by Cook et al. [26]. The quantity of supplement eaten, average weight gain and FCR were then compared between feeding groups.

### Chemical analysis

To determine if the leaf contents were taken up by the fish, fish carcasses and feeds were analysed for dry matter and crude ash according to Hilrich [27]. GE content was determined using bomb calorimetry (IKA C 7000, Janke & Kunkel IKA-Analysentechnik, Germany). CL was extracted from feeds and fish carcasses using the Soxhlet method. The extracted lipid and lipid-free fractions were analysed for carbon and nitrogen content and isotopic signatures using an Elemental Analyzer (EA, Euro Vector, HEKAtech, Wegberg, Germany) and a Isotope-ratio mass spectrometer (IRMS; Delta Plus Advantage, THERMO, Bremen, Germany), respectively. Crude protein content was calculated based on the elementary nitrogen content with a factor of 6.25.

### Bacterial challenge

After a 40 wk. feeding period, half the tanks from each feeding group were exposed to the bacterium: fish were injected intramuscularly with 100 μl of 0.9% sodium-chloride solution in which *A. hydrophila*, originally isolated from the skin lesions of clinically diseased grass carp from Northern Vietnam, had been re-suspended at a concentration of 10^8^ CFU/ml. The other three tanks in each feeding groups were used as control and were injected intramuscularly with 100 μl of a sterile solution of 0.9% sodium-chloride solution. The feeding regimes were left unaltered after infection.

Mortalities were monitored daily. To confirm *A. hydrophila* as the cause of death, the head kidney from each fish was removed and homogenized in phosphate-buffered saline (PBS) at a final concentration of 1 g/ml. The homogenates were serially diluted, plated on Columbia sheep blood agar in three replicates and incubated at 26 °C for 48 h. This produced a pure culture and the bacterial colonies were identified based on their morphology and counted. Afterwards, this identification was confirmed using the analytical profile index (Biomerieux, Marcy-l’Étoile, France) which allowed to confirm that the isolate had a profile identical to the bacterium used in the challenge.

Skin lesions were macroscopically examined and evaluated according to the criteria of Lio-Po et al. [14]. The severity of the skin lesion observable on the surface of each fish was recorded and the average grade of the skin lesions was calculated for each feeding group.

The experiment was terminated 10 d after infection, when fish were euthanatized by immersion in a solution of Tricaine Methane Sulfonate (MS222, Sigma Aldrich) at a concentration of 100 mg/l of water. Blood was collected from the caudal vein and immediately transferred to heparin-coated micro haematocrit capillaries and centrifuged at 5000 rpm for 7 min at room temperature. Anterior kidneys were sampled for bacterial isolation. The fish that were still alive at the time of termination were considered to have survived infection, as confirmed by the absence of growth from the anterior kidneys.

### Statistical analysis

Growth and feed parameters, chemical carcass composition, and isotopic signature of lipid and lipid-free fractions were analysed by factorial ANOVAs using PSPP and STATISTICA 8 (StatSoft®, Tulsa, OK, USA). Data sets were tested for homogeneity using Levene’s test and checked visually for normality. The number of dead fish in each treatment were analysed by chi-square test while the haematocrit levels, bacterial loads in the head kidney of infected fish as well as the severity of the skin lesions on their surface were evaluated by pairwise ANOVAs using SPSS 17 (IBM corporation, New-York City, USA) and PSPP (Free software Foundation). Samples were tested for normality using the Kolmogorov-Smirnov test and for homogeneity of variance using Levene’s test. Because the FCR values with supplement were non- normally distributed, the natural logarithms (Ln) were calculated for these values and confirmed to be normally distributed and homogeneous in variance using the Kolmogorov-Smirnov test and Levene’s tests. Afterwards, ANOVA was performed on these values.

## Additional files

Additional file 1: Feed consumption. Feed consumed during the experiment. This data summarise the feed consumed over the course of the experiment for each tank of fish. This data is calculated with and without taking the supplement into accounts. (XLS 75 kb)

Additional file 2: Feeding and growth. Daily feeding and total biomass. Description of data: This data present the daily recorded feeding for each tank, as well as the estimated biomass of fish in the tanks on each day. (XLSX 2111 kb)

Additional file 3: Infection this data concern the follow-up following the infection procedure. The numbers of mortality are reported alongside the skin lesions and the bacterial loads in colony forming units per ml of head kidney. (XLSX 80 kb)

## Abbreviations

*A. hydrophila*: *Aeromonas hydrophila*
CFU: Colony forming unit
CL: Crude lipid
CP: Crude protein
DM: Total dry matter
EA: Elemental analysis
FCR: Feed conversion ratio
GE: Gross energy content
IRMS: Isotope-ratio mass spectrometry
MS222: Tricaine methane sulfonate
PBS: Phosphate-buffered saline
PER: Protein efficiency ratios
RPM: Rotations per minutes
SGR: Specific growth rate
